# Protein Scaffolds Can Enhance the Bistability of Multisite Phosphorylation Systems

**DOI:** 10.1371/journal.pcbi.1002551

**Published:** 2012-06-21

**Authors:** Carlo Chan, Xinfeng Liu, Liming Wang, Lee Bardwell, Qing Nie, Germán Enciso

**Affiliations:** 1 Department of Mathematics, Center for Mathematical and Complex Biological Systems, Center for Complex Biological Systems, University of California, Irvine, California, United States of America; 2 Department of Mathematics, University of South Carolina, Columbia, South Carolina, United States of America; 3 Department of Mathematics, California State University, Los Angeles, California, United States of America; 4 Department of Developmental and Cell Biology, School of Biological Sciences, University of California, Irvine, California, United States of America; ETH Zurich, Switzerland

## Abstract

The phosphorylation of a substrate at multiple sites is a common protein modification that can give rise to important structural and electrostatic changes. Scaffold proteins can enhance protein phosphorylation by facilitating an interaction between a protein kinase enzyme and its target substrate. In this work we consider a simple mathematical model of a scaffold protein and show that under specific conditions, the presence of the scaffold can substantially raise the likelihood that the resulting system will exhibit bistable behavior. This phenomenon is especially pronounced when the enzymatic reactions have sufficiently large *K_M_*, compared to the concentration of the target substrate. We also find for a closely related model that bistable systems tend to have a specific kinetic conformation. Using deficiency theory and other methods, we provide a number of necessary conditions for bistability, such as the presence of multiple phosphorylation sites and the dependence of the scaffold binding/unbinding rates on the number of phosphorylated sites.

## Author Summary

The modification of a protein at multiple sites can result in a number of interesting behaviors at the cellular level, such as all-or-none responses to an external input, or two different stable cellular states in otherwise identical environments. Such behaviors can aid in many different forms of cellular decision-making, e.g., cell differentiation or cell division. In this paper, we show that bistable behavior can be greatly enhanced by the presence of a scaffold protein, which binds to the substrate protein and either relocates it or otherwise affects the action of the modifying enzymes. The scaffold protein substantially widens the range of parameters for which bistability is observed when, a key descriptor of enzymatic activity, assumes medium to large values found in a majority of enzymes. Indeed, when  was greater than the concentration of the target substrate, bistability was never observed in the absence of a scaffold. In addition to extensive computational work, we also carried out a mathematical analysis of a simplified system in order to identify the conditions under which bistability is possible. We conclude that scaffold proteins can be a simple yet very useful addition to multisite protein systems when bistability is advantageous.

## Introduction

Protein phosphorylation is a ubiquitous form of post-translational modification [1]. Since covalently-bound phosphate groups are strongly hydrophilic and negatively charged, they can activate or inhibit a protein by changing its conformation or the way it interacts with other proteins [2], [3]. Phosphorylation is a key element of biological regulatory processes including signal transduction, gene regulation, the cell cycle, and protein degradation [4].

Multisite phosphorylation is also a very common occurrence. For example, Epidermal Growth Factor (EGF) receptor activation involves phosphorylation at multiple tyrosine residues by another EGF receptor [1]. Also, many proteins have a surprisingly large number of phosphorylation sites. For example, nine phosphorylation sites were identified in the cyclin-dependent kinase inhibitor Sic1 [5], more than 30 sites in EGF Receptor (EGFR) and several dozen in p53 [6].

It has long been known that the presence of multiple ligand binding sites in a protein can give rise to cooperative binding through allosteric interactions between binding sites [7]. Phosphorylation-dephosphorylation reactions (and other reversible enzymatic reactions) can exhibit a related property known as *ultrasensitivity*, consisting of a steep, switch-like response of output to increasing input concentrations. *Bistability* refers to the ability of a deterministic system to have two stable steady states. This property is useful in all-or-none cell fate decisions, such as the decision to differentiate, or to progress through the cell cycle [8]. Another potential advantage of bistability is that it might allow genetically identical cells to respond heterogeneously to nearly-identical conditions [9]; this is thought to be advantageous for unicellular organisms [10]. Bistability in natural systems is often thought to result from the existence of an overt positive feedback loop [11]. More recent work with multisite phosphorylation systems, however, has revealed that bistability can occur in the absence of such a loop [12], [13], [14].

Biochemical models of multisite phosphorylation have been studied in the literature with an eye towards ultrasensitivity and bistability, see for instance Gunawardena [15]. In [16] some of us introduced scaffold proteins and showed that the presence of the scaffold strongly increased the ultrasensitive behavior of the system under various parameter conditions. Several other plausible mechanisms have also been suggested to enhance the ultrasensitive response [17], [18], [19].

In this paper, we focus on the bistability of multisite phosphorylation systems with scaffold proteins. Four mathematical models with different topology and assumptions are developed. An analytical study using deficiency theory [20], [21], [22] is carried out in search for network topologies that can support bistable behavior. Then, through systematic exploration of parameter space, we conclude that scaffold proteins substantially increase the likelihood of bistability, in the sense that a larger fraction of randomized parameter sets exhibits this property. This holds even for systems where bistability is observed without scaffold protein. On the other hand, we find patterns in kinetic parameters that are more likely to have bistability.

### Description of the model

**Figure 1:**
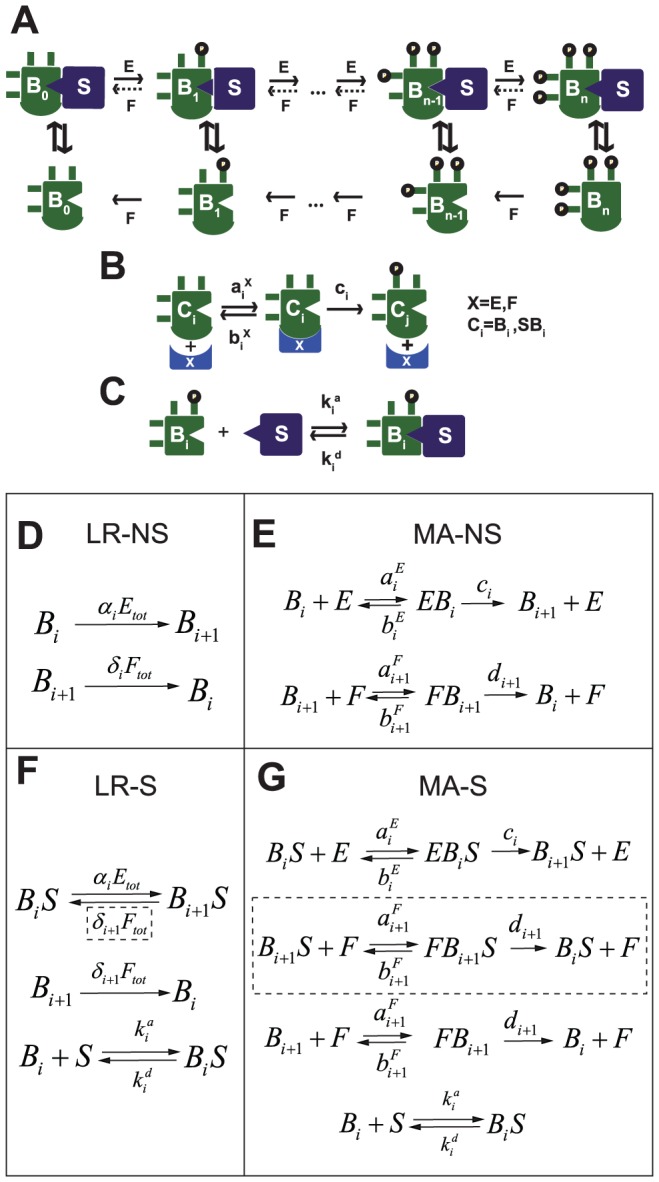
Models of n-site (de)phosphorylation of substrate 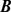 with scaffold protein 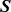. A) Phosphorylation occurs only on scaffold-bound substrates, and dephosphorylation can take place both on and off scaffold except when stated otherwise. 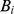 represents a protein that has been phosphorylated 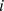 times, and 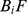 represents the scaffold-bound protein. Phosphorylation is mediated by a kinase 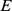, and dephosphorylation is facilitated by a phosphatase 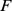. B) Full enzymatic (de)phosphorylation mechanism using standard mass action kinetics (MA). The parameters 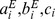 represent the on, off, and catalytic rates for the phosphorylation reaction. For the dephosphorylation reaction these rates are 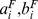, and 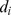, respectively. C) Mechanism for scaffold binding. The substrate binds with the scaffold to form a heterodimer that can also unbind back to its original form. The parameters 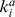 and 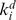 represent the on and off rates, respectively. D–G) We distinguish between models with a scaffold (S) and models with no scaffold (NS), as well as models with full mass action enzymatic reactions (MA) and models with simplified, linear enzymatic reaction rates (LR). This gives rise to the four models MA-S (D), LR-S (E), MA-NS (F), and N LR-S (G). The reactions in the dotted squares are omitted when dephosphorylation only takes place off-scaffold.

The multisite (de)phosphorylation system is modeled using a standard sequential mechanism (Figure 1A). To introduce the scaffold we allow for reversible binding between the scaffold protein 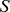 and the substrate 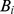 with *i* phosphorylated sites, to form the complex 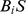 (Figure 1C). We allow phosphorylation to take place only for the scaffold-bound substrate, due to the fact that scaffolds accelerate substrate phosphorylation either by tethering the kinase and the substrate in proximity to each other, or by allosterically activating the kinase or the substrate [23], [24]. The degree of rate acceleration by scaffold proteins can be as much as 10,000 fold [23].

With regard to dephosphorylation, it has been proposed that some scaffold proteins may protect bound proteins from the action of phosphatases [25], [26], while other scaffold proteins actually recruit phosphatases in addition to kinases [27]. We assume by default that dephosphorylation takes place equally on and off the scaffold, but we will also consider cases where phosphatases act exclusively off the scaffold.

To quantify the dynamics of multisite phosphorylation, we have explored two types of commonly used mechanisms: full mass action kinetics (MA) [12], [13], [14], and simplified linear enzymatic rates (LR). In the linear rate model LR, the rates of flux of 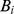 through phosphorylation and dephosphorylation are given by 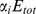 and 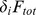 respectively, where 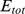 and 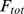 are the total kinase and phosphatase concentrations (Figure 1D,F). In the full model MA, the free kinase concentration 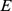 is distinguished from the total kinase concentration 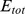, and phosphorylation follows a standard Michaelis-Menten mechanism of complex formation using 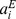, 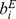, and 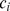 as the on, off, and catalytic rates, respectively. Similarly for the dephosphorylation mechanism (Figure 1B,E,G). The full model has many more variables, parameters, and nonlinear reaction terms than the simplified LR model for a given total number of sites, which in practice means that LR is more amenable to mathematical analysis [16]. In fact, it is known that in the absence of a scaffold the LR model always results in a unique steady state, while the full model can support multistability [12], [13], [14]. We termed the simplified model without scaffold as “LR-NS” (Figure 1D), the simplified model with scaffold as “LR-S” (Figure 1F), the full model without scaffold as “MA-NS” (Figure 1E), and the full model with scaffold as “MA-S” (Figure 1G).

It is worth pointing out that a distributive mechanism is assumed for (de)phosphorylation on scaffold, that is, that the enzymes tend to unbind from the substrate after each modification. There is evidence that some scaffold proteins may behave in this way. For example, the Ste5 scaffold protein binds weakly to its associated kinases [28], and it has even been hypothesized that one of those kinases (Ste7) may be frequently ejected from the Ste5 as a result of feedback phosphorylation [29]. Similarly, human MEK1 protein, when bound to the KSR scaffold protein, is thought to be phosphorylated by an (unbound) trans-acting homodimer of the RAF kinase [30]. If a kinase were to remain bound to the scaffold through multiple, processive phosphorylation events, however, this would be expected to reduce the propensity of the scaffold to promote bistability.

## Results

### Monostable topologies

Before investigating the parameter patterns of bistable multisite (de)phosphorylation systems with scaffold, we first explore network topologies that exclude bistability regardless of kinetic parameter values. To this end, we employ the deficiency theory developed by Feinberg and others [20], [21], [22], and we restrict our attention to the simplified linear rate model with scaffold, LR-S.

The deficiency theory of chemical reaction networks is able to predict under certain circumstances that a given system is incapable of having multiple steady states, regardless of the parameter values used (assuming fixed total protein concentrations). In order to do this, it only makes use of qualitative graphic-theoretic properties of the network, such as the number 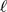 of connected components in the reaction diagram, and the number 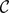 of nodes in this diagram, called *complexes*. For instance, the reaction network 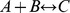, 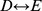 has 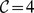 complexes (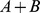, 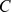, 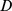, and 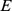) and 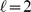 connected components. The *deficiency* of the network is defined as 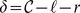, where 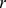 is the rank of the stoichiometry matrix. The most widely used result in the theory is the Deficiency Zero Theorem, which states that if 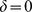 and every connected component is strongly connected (such as in the simple example above), then multistability is impossible, regardless of parameter values. That theorem is the basis for several of the results in this analysis. Please refer to Section 1 in Text S1 for details on the proofs of all results.

**Figure 2:**
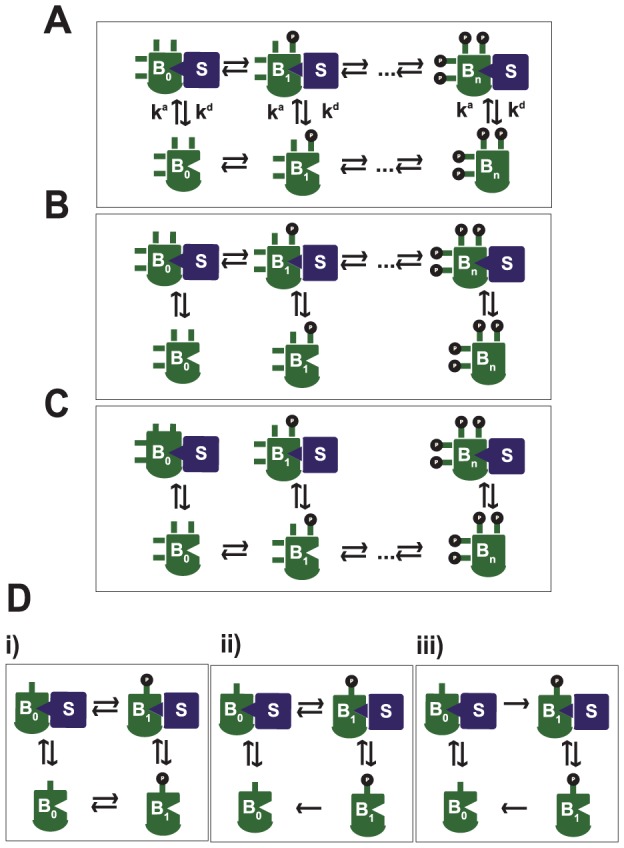
Network topologies that only support monostability under the linear model (LR-S). A) Phosphorylation and dephosphorylation take place equally on- and off-scaffold, and the scaffold binding parameters 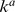 (as well as 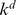) are equal across all phosphoforms 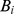. B) Phosphorylation and dephosphorylation happen only on scaffold-bound substrates. C) Phosphorylation and dephosphorylation take place only on scaffold-unbound substrates. D) Single phosphorylation site, 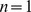. Di): phosphorylation and dephosphorylation both on- and off-scaffold. Dii): same as Di), but without phosphorylation off-scaffold, Diii) same as Dii), but without dephosphorylation on scaffold.

If the scaffold association and dissociation rates 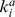 and 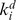 are independent of the phosphorylation state of the system, i.e., 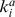 and 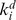 are constant for all values of 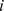, then there cannot be multiple steady states (Figure 2A). In other words, to achieve multistability, the scaffold binding mechanism must be related to, or affected by, the phosphorylation state of the substrate. This is consistent with the finding in [16] that scaffold sequestration rates need to vary with the phosphorylation state, in order to affect the ultrasensitive behavior of the system.

*Proof sketch*: define the variables 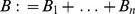, 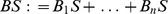. Since the phosphorylation state is irrelevant for the scaffold binding and unbinding reactions, the variables 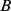, 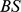 are the solutions of system 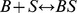.

If phosphorylation and dephosphorylation only take place for scaffold-bound substrates, then the system can only have one steady state (for given total concentrations of the substrate, enzymes and scaffold) (Figure 2B). The same conclusion holds if phosphorylation and dephosphorylation only take place away from the scaffold (Figure 2C). In order to allow for bistability, both the scaffold-bound and the scaffold-unbound proteins must have access to at least one type of enzyme – the kinase or the phosphatase.

*Proof sketch*: the two statements follow directly from the Deficiency Zero Theorem – however notice that Figure 2B and 2C are only diagrams in that the complex 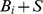 is shortened as 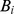. In Figure 2B, 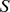 can be easily included as necessary and 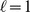, 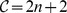, 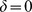. In Figure 2C, including 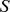 in the scaffold binding reactions but not the phosphorylation reactions forces to rewrite the graph as shown in Section 1.2 of Text S1, and 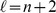, 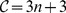, 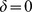.

We point out that even though the model in Figure 2C is always monostable, this particular topology has shown to be highly ultrasensitive for some parameter values [16], which underscores the difference between ultrasensitive behavior and bistability.

Even in the presence of a scaffold, a substrate with a single phosphorylation site is incapable of producing bistable behavior for several possible network configurations (Figure 2D). This result provides evidence that if the kinase and the substrate both remain bound to the scaffold long enough, on average, for the kinase to catalyze two or more phosphorylation events in a processive manner, then the propensity for scaffold-driven bistability will be reduced. The proof for all configurations given in Figure 2D is given in Section 1.4 of Text S1, and it is based on exploring the signs of the entries in the stoichiometric matrix as well as all its square submatrices.

### Kinetic constraints for bistability

We take a closer look into the parameter values of the linear rate scaffold system LR-S, in search for patterns that might make bistability more likely. We simulate this system using a large set of phosphorylation, dephosphorylation, and scaffold binding and unbinding parameters. In particular, we randomly sample each of those parameters over a range of several orders of magnitude, consistent with experimental measurements [13].

**Figure 3:**
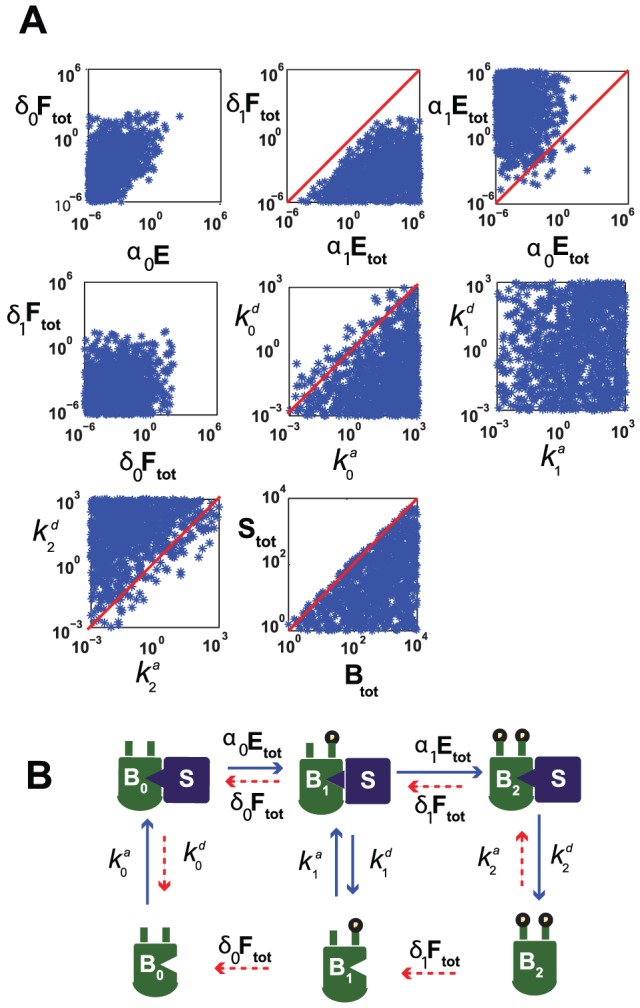
Kinetic constraints for multistability with linear (de)phosphorylation rates with scaffold binding (LR-S) for two phosphorylation sites (n = 2). A) After each randomization of all the parameters the system is tested for bistability. If the test is positive one star is placed on each of the graphs in order to describe the parameter set. B) Cartoon depiction of the specific kinetic behavior, based on the parameter selection plot in (A). The dashed red arrows represent weaker interactions as measured in (A).

For two-site (de)phosphorylation systems with scaffold, our numerical simulations suggest (Figure 3A): (1) for every single bistable system found, the rate of phosphorylation from 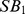 to 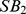, 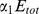, is larger than the rate of dephosphorylation per unit phosphatase from 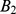 to 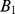 (and from 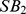 to 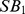), 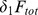; (2) 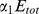 is also almost always larger than 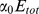, the rate of phosphorylation from 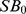 to 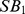; (3) the scaffold dissociation constant is low for 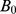 (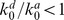) and high for 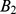
(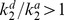); (4) the total substrate concentration 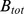 is larger than the total scaffold concentration 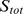.

In summary, the above features in parameters indicate a fast flow 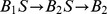, together with a small flow out of 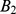, ensuring that there is an accumulation of phosphorylated protein in the scaffold-unbound state (Figure 3B). Similarly, the scaffold-bound, unphosphorylated protein 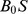 accumulates due to a low scaffold dissociation rate. This configuration can give rise to multiple steady states, where in fact most of the protein accumulates at either 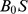 or 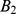 (data not shown).

It is remarkable that this is the *only* common conformation giving rise to bistability under the chosen parameter regime. Due to the mass conservation of total substrate, a conformation in which all variables are present in either high or low concentrations is precluded. The fact that we do not allow for phosphorylation off scaffold also breaks some of the possible symmetries. It is also significant that bistability is rarely observed when 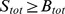. In fact, for large relative amounts of 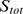, it holds that 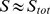 and the system becomes approximately linear. Hence in the limit it cannot have two discrete stable steady states.

It is worth pointing out that although the cell membrane was considered a suitable scaffold for ultrasensitive behavior in [16], it may not itself be a good scaffold for bistable behavior, since 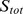 must be limiting for bistability. Given that the cell membrane has a relatively large surface area, it is not likely that binding sites on the membrane will be saturated by a given membrane-binding regulatory protein. Thus, under the hypotheses of this model, employing the plasma membrane as a scaffold would be unlikely to aid in the promotion of bistability. On the other hand, the scaffold may well be a membrane-bound protein available in limited concentration. This effectively recruits the substrate onto the membrane while limiting the total amount of scaffold.

### Scaffold binding can strongly enhance multistability for *K_M_*>*B_tot_* or *K_M_>1 µM*

**Figure 4:**
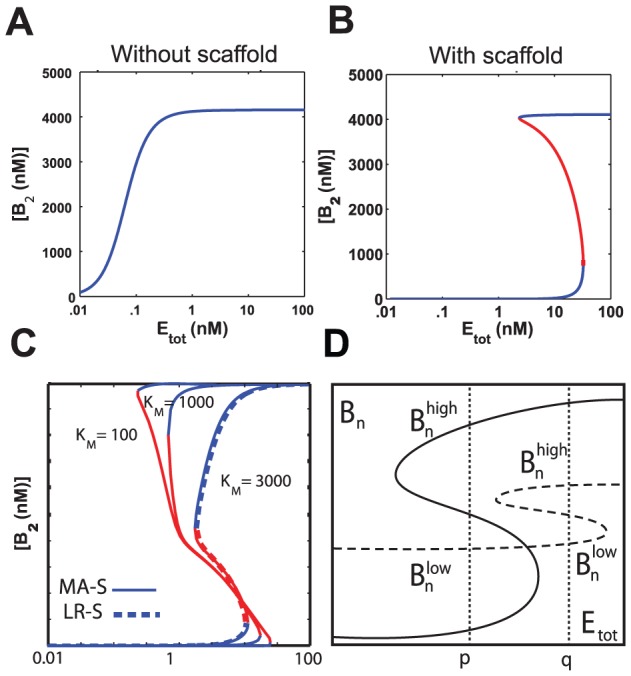
Dose responses and bistability. A) Dose response curve without scaffold binding (MA-NS) exhibits one steady state for any given input. B) The full model MA-S with scaffold binding, using the same parameter set and additional scaffold parameters, exhibits multiple steady states for certain inputs. See Table S1 for parameter values. C) Comparison of LR-S and MA-S models. The dashed curve represents the dose response for LR-S, and the solid curves the dose response of MA-S for corresponding parameters and increasing 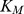 values. See Table S2 for parameter values. D) Comparison of the fold ratio of steady states for two hypothetical dose response curves. For the dotted line, the ratio 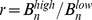 for the input 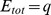 is relatively small compared to the fold ratio 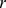 of the solid line for 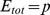.

We now consider the full mass action (MA) models and to what extent the addition of a scaffold facilitates bistable behavior. First we show that, at least for some sets of parameters, a scaffold allows a monostable multisite system to become bistable. By examining the dose-response curve as a function of the total kinase concentration 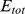, no bistability is observed for the system MA-NS (Figure 4A). However, in the presence of the scaffold, with the same phosphorylation parameters (except now phosphorylation takes place only on the scaffold), the response curve presents bistability for a range of values of 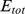 (Figure 4B). We randomize every parameter in the system over several orders of magnitude (see the Methods section for full ranges), in order to find whether this behavior is typical. One preliminary result was that for 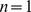 no bistable behavior was found computationally for either MA-NS or MA-S, consistent with the theoretical findings for LR-NS and LR-S in the section on monostable topologies. Therefore in the following we focus on systems with multiple sites.

In Figure 4C, we compare the behavior of the simplified system LR-S to that of MA-S, and we find that the dose response of both systems becomes very similar for large values of the Michaelis constant 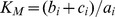. This parameter is essential in the quantitative study of enzymes and constitutes the substrate concentration at which the enzymatic reaction takes place at half the maximal rate. It is important to note that most enzymes have been experimentally found to have a 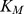 between 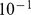 and 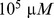 (Section 8.4 in [31]). When 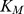 is relatively large in a given enzymatic reaction, the flow rate from the substrate 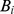 to the product 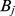 can be estimated to be 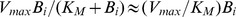 [32], i.e. the detailed mass action model MA becomes similar to the linear rate model LR (both in the presence and in the absence of a scaffold). See also a more detailed mathematical analysis in Section 3 of Text S1.

We are particularly interested in bistable behavior with a significant distance between steady states. To this end, we define 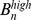 and 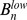 as the highest and lowest stable steady state values of 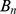 in the case that multiple steady states exist. We restrict our definition of bistability to the case where 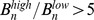. This definition is biologically relevant. Imagine a biological circuit with two stable steady states that are close to each other. This system is likely to have similar properties as one with a single stable steady state. It is well known that bistability can give rise to cell differentiation or other types of cellular decision-making. The underlying premise is that one of the proteins in the system, say, the most phosphorylated version of the substrate, is responsible for activating a downstream response that triggers one of the two possible cellular behaviors. If this protein is not present in sufficient concentration, the other cellular behavior should result. Therefore in practice, bistability by itself is not enough, but the two different steady states (or at least the key active proteins) should be sufficiently different from each other (Figure 4D).

In order to systematically compare different systems, we classify the models according to the 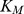 value of the different enzymatic reactions. Thus enzymatic parameters are chosen randomly in such a way that all the individual 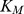 values lie within a specified range of one order of magnitude. For 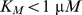, the system tends to be bistable even without the addition of a scaffold, and adding a scaffold decreases the probability for bistability (Table 1).

**Table 1:** Percentage of bistable parameter sets (MA) for increasing 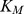 values.

			% Likelihood of bistability for given 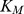 Range 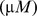			
	Off scaffold	On scaffold	(0.1,1)	(1,10)	(10,100)	(100,1000)
n = 2	ph./deph.		2.8	0	0	0
	deph.	ph./deph.	5.6	3.4	3.6	3.0
	deph.	ph.	4.0	7.0	7.0	5.4
n = 3	ph./deph.		8.2	0.8	0	0
	deph.	ph./deph.	8.8	5.8	7.0	7.6
	deph.	ph.	9.4	10.6	12.8	14.8
n = 4	ph./deph.		11.4	1.6	0	0
	deph.	ph./deph.	12.4	8.6	10.4	14.6
	deph.	ph.	15.2	13.0	18.4	21.0
n = 5	ph./deph.		14.6	2.2	0	0
	deph.	ph./deph.	16.2	13.2	15.6	18.4
	deph.	ph.	18.6	20.4	25.6	29.8

The percentage of parameter sets generating a bistable multisite phosphorylation system with or without scaffold is described for 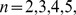 using full mass action kinetics (MA) and classified according to the 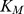 value of the enzymatic reactions. The 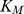 vary from 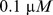 to 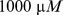 and are grouped in 10-fold regimes. Each entry in the table was created using 500 independent parameter sets. In order to ensure a sufficient difference between the steady states, we assume a fold ratio larger or equal than 5 between the largest and the smallest steady state of 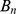, i.e.\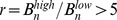.

However, for 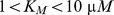, the likelihood of bistability in the scaffold model (13.2% for *n = 5*) is several times that of the model without a scaffold (2.2%). For 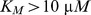, the effect of adding a scaffold becomes much more pronounced. Simulations based on a set of 500 randomly chosen parameters for each entry in Table 1 indicate that in the absence of a scaffold the system is monostable for such 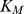. We next increase the number of randomly chosen parameters to 100000 for the range of 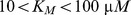, without scaffold and no bistability is found. Remarkably, if a scaffold is considered in the same circumstances the probability of bistability leaps up to 18.4% for *n = 5*, which is significant considering that the on and off rates as well as the total protein concentrations are randomly varied over several orders of magnitude. If the phosphatase acts only off the scaffold, the probability for bistability further increases to 29.8%. Similar results as in Table 1 are found when the assumption of a sufficient ratio between the steady states is dropped, see Table S3. Also, analogous results were found when the off-scaffold phosphorylation rate is low but nonzero as well as when all 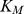 lie within ranges of two orders of magnitude (data not shown).

It should be noted that the value of 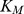 is often important only with respect to the concentration of the corresponding substrate. Here we have assumed ranges for 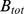 from 1 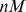 to 10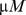 (see the Methods section), and it is possible that a relevant measure for the results in the table is 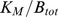. In Table 2, we repeat the same analysis as in Table 1 but classifying the parameter sets by this ratio instead of 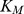. We find that whenever 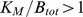, that is when 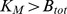, there is no bistability without scaffold, but the addition of a scaffold does allow a significant likelihood for bistability.

**Table 2:** Percentage of bistable parameter sets (MA), for increasing 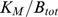 values.

			% Likelihood of bistability for given 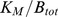 Range				
	Off scaffold	On scaffold	(0.1,1)	(1,10)	(10,10^2^)	(10^2^,10^3^)	(10^3^,10^4^)
n = 2	ph./deph.		2.2	0	0	0	0
	deph.	ph./deph.	6.8	4.6	4.0	6.4	2.8
	deph.	ph.	4.2	2.8	4.0	3.0	4.8
n = 3	ph./deph.		6.2	0	0	0	0
	deph.	ph./deph.	5.0	7.4	8.4	9.0	7.8
	deph.	ph.	9.8	9.0	11.8	11.6	9.4
n = 4	ph./deph.		10.0	0	0	0	0
	deph.	ph./deph.	13.6	8.2	10.8	14.4	8.0
	deph.	ph.	13.0	12.2	15.8	16.4	17.4
n = 5	ph./deph.		8.6	0	0	0	0
	deph.	ph./deph.	13.6	11.2	14.4	17.2	10.2
	deph.	ph.	17.4	16.4	19.0	21.2	19.2

The percentage of parameter sets generating a bistable system is described for 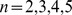 as in Table 1, but classified according to the ratio of 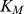 to the total substrate concentration, 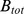. Notice that for a ratio 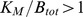 no bistability is found without scaffold. That is, whenever 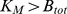 we found that bistability is only possible in this model after the addition of a scaffold.

Notice also that these results hold regardless of the dimensionality of parameter space or of the geometry of the set of bistable parameters, since we are merely measuring the proportion parameter sets that yield bistable systems. As a matter of reference, if the fraction of bistable parameter sets under given conditions is around 10% and 500 samples are taken, one can expect about 1.3% of standard deviation between the sampled result and the actual fraction.

## Discussion

In cellular signal transduction, multiple, consecutively-acting components of a signaling pathway are often physically organized into complexes by scaffold proteins. Here, by exploring various models of multisite (de)phosphorylation with scaffold, we conclude that under the following specific conditions the presence of a scaffold can enhance bistability of multisite phosphorylation systems.

1. **Non-processive multisite substrate phosphorylation.** The signaling proteins that bind to scaffolds are often phosphorylated at multiple sites and believed to act in a non-processive manner, for instance in the case of the MAPK cascade (MAP3K, MAP2K and MAPK). Many such proteins are organized by scaffold proteins [33]; furthermore, the activity of each kinase is regulated by phosphorylation of two or more distinct sites [34].
2. **A substrate concentration in the same order of magnitude or higher than the scaffold concentration.** This assumption is also reasonable; for instance, in the case of the yeast mating MAPK cascade, several measurements indicate that the cellular concentrations of the Ste5 scaffold and its associated kinases Ste11 and Ste7 are all around 30–50 nM (i.e., ∼700 molecules/cell), and the ultimate substrate of Ste5 scaffolding, Fus3, has a cellular concentration that is at least 5-fold greater [28], [35], [36].
3. **Kinases with a relatively high** 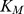 **value.** For such enzymatic reactions the kinase-substrate complex is relatively transient. This assumption can be explained on the grounds that high 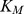 values (relative to substrate concentrations) tend to make a system more linear. In the absence of a scaffold, such a linear system cannot exhibit multistable behavior. In the case of low 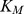, the flow rates for an enzymatic reaction can be approximated by a constant proportional to the total enzyme. Thus the steady state substrate distribution depends subtly on the total enzyme and phosphatase concentrations, leading to zero-order ultrasensitivity [37]. The bifurcation graph using 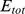 as a bifurcation parameter is likely to be highly ultrasensitive, which might make it more likely that the system is already bistable for similar parameters without the need for a scaffold [13]. This is evidenced on the first column of Table 1.
4. **A scaffold-substrate dissociation constant that varies with the substrate phosphorylation state.** A possible implementation of this assumption is through a bulk electrostatic mechanism for substrate binding. If the scaffold is naturally negatively charged near its binding domain to the substrate, then the presence of negatively charged phosphorylations at the scaffold might prevent its binding and accelerate its unbinding. In principle, this can take place for a multisite scaffold protein in the absence of allosteric behavior. For instance, in the yeast pheromone-response pathway, protein Ste5 (itself a scaffold protein but here viewed as a substrate) binds to the membrane in part due to bulk electrostatic interactions that are modified by multisite phosphorylation [38].

Notice that certain relations among the various parameters are also consistently preserved. For instance, the larger phosphorylation rate for the second site suggests an allosteric behavior between the substrate and kinase.

Several different models of bistability in protein networks are described in [39]. In [40], protein sequestration is considered as a means to obtaining bistability in an apoptosis network. Another approach was carried out in [41] for the MAPK system, where the activity of MEK is inhibited by unphosphorylated ERK acting as a scaffold. These systems are similar in spirit to this work, although they likely exploit a different mechanism for bistability. For instance, in [41] bistability takes place largely because the substrate is allowed to phosphorylate the scaffold and alter its binding activity, a key feedback component that we do not assume here. Also in that model the scaffold must be in excess of the substrate for bistability ([41], Figure 3A), whereas in our system we have the opposite requirement. See also the work in [42], where a 25-fold parameter variation analysis is carried out for a MAPK model to determine the likelihood of behaviors such as bistability and oscillations.

Another important aspect to consider in these chemical reaction systems is the effect of noise and stochastic behavior. If chemical reactions are allowed to take place in a non-deterministic way, the variables in a bistable system might switch spontaneously from one steady state to another. Here the fold-change measure introduced in the Results section is again useful: if the distance between the two steady states is increased, one can expect in general that the frequency of such spontaneous events is reduced. To the extent that the addition of a scaffold increases this distance, it may reduce the effect of noise. Also, we have found, for LR-S, that bistability is in a sense characterized within a certain parameter regime. If parameters are changed due to stochastic effects, bistability will tend to be preserved as long as the parameters remain within that regime. In that sense bistability in LR-S can be described as robust with respect to parameter noise.

Notice that the simulations in Table 1 suggest that zero-order ultrasensitivity isn't just a mechanism for bistability in the traditional non-scaffold system, but the only such mechanism. This is because low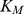 values (or low 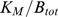 ratios) seem to be necessary for bistability. Also, the results in Figure 3 suggest that ligand binding, as opposed to phosphorylation, could provide a framework for bistability using scaffolds. Assuming that the ligand is in high concentration, a simple model of multisite ligand binding would look very much like LR-S and the same analysis would likely apply.

We have concluded that adding a scaffold has a large likelihood of turning a monostable multisite system into a bistable one, for large 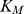-to-substrate ratio. The intuition behind this result can be described as follows. Recall that for large values of 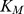 the MA system resembles the LR system, with and without scaffold respectively. Suppose that a parameter regime is such that the 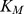 are large, and that the relationships in Figure 3B are satisfied. Then LR-S is likely to be bistable, and the corresponding system MA-S is likely bistable as well since it resembles LR-S. On the other hand, LR-NS must be monostable because it is fully linear, and MA-NS is likely monostable too since it resembles LR-NS. Therefore for such a regime MA-S is much more likely to be bistable than MA-NS. This conclusion is further justified mathematically in Section 3 of Text S1.

Scaffolds typically do not possess any enzymatic activity themselves, but facilitate signaling between their bound components. One way in which they are thought to do this is by tethering their ligands in close spatial proximity to each other [24]. Another mechanism by which scaffolds can enhance signal transmission is to induce an allosteric conformational change in a bound substrate that reveals target residues, as exemplified by yeast Ste5 (scaffold) unlocking Fus3 (substrate) for phosphorylation by Ste7 (kinase) [23], and human KSR (scaffold) unlocking MEK (substrate) for phosphorylation by RAF (kinase) [30]. In addition to speeding up certain rates, scaffolds may also slow down the rates of other enzymatic reactions by blocking the access of certain enzymes (e.g., phosphatases) to bound ligands. Regardless of the precise mechanism by which they act, scaffolds generally exhibit two key properties examined in this work: sequestration and rate partition. By sequestration, we mean that the scaffold-bound population is separated from the unbound (e.g., cytoplasmic) population, essentially creating two different compartments. Of course, if reaction rates and enzyme/substrate concentrations are the same in these two compartments, the scaffold will essentially be inert. Thus, rate partition –the ability of the scaffold to speed up or slow down the rate of enzymatic reactions by one of the mechanisms described above– is also crucial for forming an effective scaffold.

The mathematical model of scaffolding employed herein features these two key elements of sequestration and rate partition. Sequestration is achieved in our model by accounting for the second order mass action binding of scaffold and substrate. Rate partition is achieved by allowing different rates of substrate modification depending on whether the substrate is bound to the scaffold or not. Our simple model does not incorporate other potentially interesting features of scaffold-mediated signaling, such as combinatorial inhibition, processive on-scaffold phosphorylation, and multi-tier scaffolding (our model just has two tiers: a single kinase and its substrate). For other theoretical treatments of scaffold action, the reader is referred to the following references: [25], [26], [43], [44], .

There has been considerable interest in understanding how common biochemical modules and motifs can be flexibly tuned to achieve a variety of desired outcomes [48], [49], [50], [51], [52]. The work presented here can be viewed as a contribution to this theme. For instance, if bistability were a desirable (pro-fitness) performance objective during an evolutionary trajectory, then a viable evolutionary strategy might be either a low-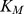 multisite phosphorylation module, or a high-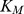 scaffolded multisite phosphorylation module. On the other hand, if multistability were to be avoided, then there are still multiple ways that a module might have evolved, either with or without scaffolding, so that other desirable performance objectives (e.g., speed, amplification, specificity, etc) might be maximized.

## Methods

Throughout the computational modeling, we used mass action kinetics to construct the systems of differential equations associated with each individual model. Given a parameter set, in order to test for bistability we first reduced the problem to a 3-variable system of equations involving 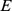, 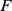, and 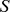, generalizing the approach described in [13] for scaffold systems; see Section 2 in Text S1 for details of this reduction for each type of model. Solutions of the reduced system were then found using Newton's Method with multiple different initial conditions for the MA models, and using polynomial numerical solvers for the LR models. Even though 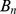 was used as the *de facto* output, we verified in hundreds of independent trials that the system is only bistable if 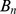 itself admits multiple stable steady states.

A key aspect of the analysis is the choice of random parameter sets over several orders of magnitude. Rates of substrate binding to an enzyme or scaffold are normally in the range of 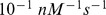 to 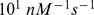 [53]. Off-rates can vary more widely depending on specificity, and they are assumed here to range from about 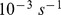 to 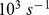 [54]. For simplicity, we choose all rate constants 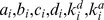, as well as 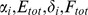 in the LR systems, between 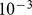 and 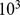 in these respective units. Total protein concentrations 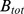, 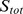 were chosen from the range 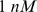 to 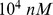. For the MA system, 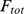 was chosen from 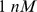 to 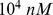, and 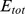 was used as a variable to plot a dose response curve as in Figure 4A,B, with values ranging from 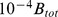 to 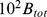. Within this range, 50 values of 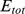 were sampled logarithmically (i.e. 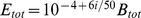, 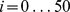) and for each value the steady states of the system were computed to create the dose response. It was determined for LR-S in Figure 3A that bistability is not found in practice for 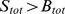, therefore to optimize the results in Table 1 and Table 2 we assumed 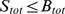. For the same reason, we restricted the ratios of the scaffold binding and unbinding parameters according to the results of Figure 3A, i.e. 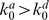, 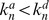. These restrictions are relatively mild considering the wide range used for each parameter.

All parameters were chosen under a logarithmic distribution—that is, using a uniform distribution for their natural logarithm. For the tables, in order to ensure that all 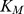 values lie within a certain range, we generated the individual rate constants as described above, and if any 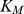 was outside of the range then the parameters were randomized once more until all 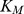 were in the desired interval.

## Supporting Information

Table S1 **Parameter set for** **Figure 4A and 4B** **.** Under these parameter values the system MA-S is bistable but MA-NS is not.
(PDF)

Table S2
**Parameter sets for** **Figure 4C** **.** Derived using the analysis in Text S1, Section 2.3, the following are the parameter sets used in Figure 4C, such that as 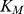 becomes larger, the dose response curve for MA-S system is approximately that for LR-S.(PDF)

Table S3
**Probability of bistable behavior for arbitrary fold ratio.** In Table 1, the percentage of parameter sets producing bistability is described for 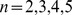, and for different 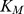 (or 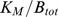) ranges, assuming a fold ratio larger or equal than 5 between the largest and the smallest steady state of 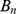, i.e.\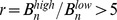. In this table we relax the last assumption and allow for an arbitrary difference between the multiple steady states. In order to ensure that the steady states found are actually different, we allow for a nominal error margin and require a fold ratio 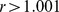. Each entry in the table corresponds to 500 independent sample simulations. The parameter sets are conditioned with the restrictions described in the Methods section, namely 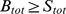, 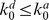, and 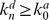.(PDF)

Text S1
**In this supplementary text we provide more information on the mathematical analysis of the various models involved.** In Section 1, we describe the concept of deficiency including a precise statement of the Deficiency Zero Theorem, then proceed to prove several of the theorems stated in the Results section of the manuscript. We also describe and apply another tool used to prove the non-existence of bistability, the concept of sign determined systems. In Section 2 we characterize the steady states of each of the four models as the solutions of two and three dimensional algebraic equations. In Section 3 we provide a detailed mathematical analysis of the idea that as 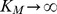, the MA systems (with or without scaffold) are approximated by the respective LR models.(PDF)
