# Supplementary material for: Protein Scaffolds Can Enhance the Bistability of Multisite Phosphorylation Systems
Source: PLoS Comput Biol. 2012 Jun 21;8(6):e1002551. doi: 10.1371/journal.pcbi.1002551 (PMC3380838; doi:10.1371/journal.pcbi.1002551)
Supplement: Table S1 — Parameter set for Figure 4A and 4B . Under these parameter values the system MA-S is bistable but MA-NS is not. (PDF) [file pcbi.1002551.s001.pdf]

.

Table S1: Parameter set for Figure 4A,4B

The following is the set of parameters used in Figure 4A and 4B, i.e. they are parameter values such that MA-S is bistable but MA-NS is not.

		$i = 0$	$i = 1$	$i = 2$
a_i^E	$nM^{-1}s^{-1}$	10^{-3}	4×10^{-2}	–
b_i^E	s^{-1}	50	4×10^{-3}	–
a_i^F	$nM^{-1}s^{-1}$	–	4×10^{-3}	10^{-3}
b_i^F	s^{-1}	–	300	50
c_i	s^{-1}	10^{-2}	10^3	–
d_i	s^{-1}	–	10^{-2}	9
$k_{M,i}^E$	nM	50010	25000.1	–
$k_{M,i}^F$	nM	-	75002.5	59000
k_i^a	$nM^{-1}s^{-1}$	4×10^{-1}	10	6
k_i^d	s^{-1}	2×10^{-3}	90	10^3
Concentrations	nM	$F_{tot} = 1$	$B_{tot} = 4 \times 10^3$	$S_{tot} = 50$
