# Supplementary material for: Protein Scaffolds Can Enhance the Bistability of Multisite Phosphorylation Systems
Source: PLoS Comput Biol. 2012 Jun 21;8(6):e1002551. doi: 10.1371/journal.pcbi.1002551 (PMC3380838; doi:10.1371/journal.pcbi.1002551)
Supplement: Table S2 — Parameter sets for Figure 4C . Derived using the analysis in Text S1, Section 2.3, the following are the parameter sets used in Figure 4C, such that as becomes larger, the dose response curve for MA-S system is approximately that for LR-S. (PDF) [file pcbi.1002551.s002.pdf]

Table S2: Parameter sets for Figure 4C

Using the analysis in Text S1 Section 2.3, the following are the parameter sets used in Figure 4C, such that as k_M becomes larger, the dose response curve for MA-S system is approximately that for LR-S.

Parameter set of dose response for LR-S:

		$i=0$	$i=1$	$i=2$
α_i	$nM^{-1}s^{-1}$	7×10^{-2}	4×10^{-2}	–
δ_i	$nM^{-1}s^{-1}$	–	4×10^{-1}	1.3×10^{-3}
k_i^a	$nM^{-1}s^{-1}$	160	20	2×10^{-2}
k_i^d	s^{-1}	8×10^{-2}	750	450
Concentrations	nM	F_{tot}=1.87	$B_{tot} = 2 \times 10^3$	$S_{tot} = 1.2 \times 10^3$

Parameter set for MA-S dose response, $k_M \approx 100$

		$i=0$	$i=1$	$i=2$
a_i^E	$nM^{-1}s^{-1}$	6.91×10^{-1}	2.48×10^{-1}	–
b_i^E	s^{-1}	1.63×10^1	5.90	–
a_i^F	$nM^{-1}s^{-1}$	–	9.56×10^{-1}	5.80×10^{-3}
b_i^F	s^{-1}	–	1.86×10^1	4.85×10^{-2}
c_i	s^{-1}	1.83	1.13	–
d_i	s^{-1}	–	1.34×10^1	1.40×10^{-2}
$k_{M,i}^E$	nM	2.61×10^1	2.82×10^1	–
$k_{M,i}^F$	nM	-	3.35×10^1	1.08×10^1

Parameter set for MA-S dose response, $k_M \approx 1000$:

		$i=0$	$i=1$	$i=2$
a_i^E	$nM^{-1}s^{-1}$	2.78×10^{-1}	4.0×10^{-2}	–
b_i^E	s^{-1}	5.1×10^{-1}	1.32×10^{-1}	–
a_i^F	$nM^{-1}s^{-1}$	–	4.0×10^{-1}	1.3×10^{-3}
b_i^F	s^{-1}	–	9.45×10^{-1}	6.65×10^{-3}
c_i	s^{-1}	5.71×10^1	1.75×10^1	–
d_i	s^{-1}	–	3.04×10^2	3.99×10^{-1}
$k_{M,i}^E$	nM	8.15×10^2	4.37×10^2	–
$k_{M,i}^F$	nM	-	7.60×10^2	3.00×10^2

Parameter set for MA-S dose response, $k_M \approx 3000$:

		$i=0$	$i=1$	$i=2$
a_i^E	$nM^{-1}s^{-1}$	1.57×10^{-1}	4.24×10^{-2}	–
b_i^E	s^{-1}	5.38×10^2	6.92×10^1	–
a_i^F	$nM^{-1}s^{-1}$	–	9.78×10^{-1}	2.02×10^{-3}
b_i^F	s^{-1}	–	1.01×10^4	1.01×10^1
c_i	s^{-1}	4.33×10^2	1.15×10^3	–
d_i	s^{-1}	–	6.95×10^3	1.83×10^1
$k_{M,i}^E$	nM	6.18×10^3	2.87×10^4	–
$k_{M,i}^F$	nM	-	1.74×10^4	1.41×10^4
