# Supplementary material for: Protein Scaffolds Can Enhance the Bistability of Multisite Phosphorylation Systems
Source: PLoS Comput Biol. 2012 Jun 21;8(6):e1002551. doi: 10.1371/journal.pcbi.1002551 (PMC3380838; doi:10.1371/journal.pcbi.1002551)
Supplement: Table S3 — Probability of bistable behavior for arbitrary fold ratio. In Table 1, the percentage of parameter sets producing bistability is described for , and for different (or ) ranges, assuming a fold ratio larger or equal than 5 between the largest and the smallest steady state of , i.e.\. In this table we relax the last assumption and allow for an arbitrary difference between the multiple steady states. In order to ensure that the steady states found are actually different, we allow for a nominal error margin and require a fold ratio . Each entry in the table corresponds to 500 independent sample simulations. The parameter sets are conditioned with the restrictions described in the Methods section, namely , , and . (PDF) [file pcbi.1002551.s003.pdf]

Table S3: Probability of bistable behavior for arbitrary fold ratio

In Table 1, the percentage of parameter sets producing bistability is described for $n = 2, 3, 4, 5$, and different K_M (or K_M/B_{tot}) ranges, assuming a fold ratio larger or equal than 5 between the largest and the smallest steady state of B_n, i.e. $r = B_n^{high}/B_n^{low} > 5$. In the following tables we relax this last assumption and allow for an arbitrarily difference between the multiple steady states. In order to ensure that the steady states found are actually different, we allow for a nominal error margin and require a fold ratio $r > 1.001$. Each entry in the table corresponds to 500 independent sample simulations. The parameter sets are conditioned with the restrictions described in the Methods section, namely $B_{tot} \geq S_{tot}$, $k_0^d \leq k_0^a$, and $k_n^d \geq k_0^a$.

Percentage of Bistable Parameter Sets, by K_M range (arbitrary fold ratio r):

	off scaffold	on scaffold	$K_M(\mu M)$: $(10^{-1}, 1)$	$(1, 10^1)$	$(10^1, 10^2)$	$(10^2, 10^3)$
$n = 2$	ph./deph.		9.0	0.8	0	0
	deph.	ph./deph.	8.2	6.6	8.2	8.6
	deph.	ph.	8.2	9.6	10.4	9.4
$n = 3$	ph./deph.		13.2	1.2	0	0
	deph.	ph./deph.	12.2	10.4	12.2	11.4
	deph.	ph.	13.2	13.2	18.2	20.6
$n = 4$	ph./deph.		15.0	2.8	0	0
	deph.	ph./deph.	16.2	14.4	14.6	19.8
	deph.	ph.	21.0	19.0	25.2	26.0
$n = 5$	ph./deph.		21.2	3.2	0	0
	deph.	ph./deph.	22.0	18.2	20.6	21.8
	deph.	ph.	22.6	25.6	32.2	34.6

We carry out similar simulations but classify them by the values of K_m/B_{tot} instead:

Percentage of Bistable Parameter Sets, by K_M/B_{tot} range (arbitrary fold ratio r):

	off scaffold	on scaffold	K_M/B_{tot} $(10^{-1}, 1)$	$(1, 10^1)$	$(10^1, 10^2)$	$(10^2, 10^3)$	$(10^3, 10^4)$
$n = 2$	ph./deph.		15.4	9.2	2.8	0.2	0.4
	deph.	ph./deph.	8.4	6.0	8.4	6.8	6.0
	deph.	ph.	11.6	10.4	9.0	10.8	12.8
$n = 3$	ph./deph.		22.6	9.6	3.2	0.4	0.2
	deph.	ph./deph.	13.0	11.8	12.6	14.0	11.0
	deph.	ph.	17.4	15.4	17.8	19.8	13.2
$n = 4$	ph./deph.		26.8	9.4	4.0	0.8	0.6
	deph.	ph./deph.	22.6	15.6	14.8	20.2	12.6
	deph.	ph.	20.0	20.0	21.0	22.2	21.8
$n = 5$	ph./deph.		22.6	11.2	6.2	0.4	2.0
	deph.	ph./deph.	21.2	18.2	21.6	22.2	12.8
	deph.	ph.	23.0	21.2	25.4	27.8	25.0

Notice that in all cases the same conclusion is found, that even though the scaffold doesn't promote bistability for low values of K_M (or K_M/B_{tot}), after a certain threshold the likelihood of bistability is greatly enhanced by its addition.

Percentage of Bistable Parameter Sets, by K_M range (fold ratio $r > 5$):

	off scaffold	on scaffold	$K_M(\mu M)$: $(10^{-1}, 1)$	$(1, 10^1)$	$(10^1, 10^2)$	$(10^2, 10^3)$
$n = 2$	ph./deph.		2.8	0	0	0
	deph.	ph./deph.	5.6	3.4	3.6	3.0
	deph.	ph.	4.0	7.0	7.0	5.4
$n = 3$	ph./deph.		8.2	0.8	0	0
	deph.	ph./deph.	8.8	5.8	7.0	7.6
	deph.	ph.	9.4	10.6	12.8	14.8
$n = 4$	ph./deph.		11.4	1.6	0	0
	deph.	ph./deph.	12.4	8.6	10.4	14.6
	deph.	ph.	15.2	13.0	18.4	21.0
$n = 5$	ph./deph.		14.6	2.2	0	0
	deph.	ph./deph.	16.2	13.2	15.6	18.4
	deph.	ph.	18.6	20.4	25.6	29.8

Percentage of Bistable Parameter Sets, by K_M/B_{tot} range (fold ratio $r > 5$):

	off scaffold	on scaffold	K_M/B_{tot} $(10^{-1}, 1)$	$(1, 10^1)$	$(10^1, 10^2)$	$(10^2, 10^3)$	$(10^3, 10^4)$
$n = 2$	ph./deph.		2.2	0	0	0	0
	deph.	ph./deph.	6.8	4.6	4.0	6.4	2.8
	deph.	ph.	4.2	2.8	4.0	3.0	4.8
$n = 3$	ph./deph.		6.2	0	0	0	0
	deph.	ph./deph.	5.0	7.4	8.4	9.0	7.8
	deph.	ph.	9.8	9.0	11.8	11.6	9.4
$n = 4$	ph./deph.		10.0	0	0	0	0
	deph.	ph./deph.	13.6	8.2	10.8	14.4	8.0
	deph.	ph.	13.0	12.2	15.8	16.4	17.4
$n = 5$	ph./deph.		8.6	0	0	0	0
	deph.	ph./deph.	13.6	11.2	14.4	17.2	10.2
	deph.	ph.	17.4	16.4	19.0	21.2	19.2
