# Supplementary material for: Protein Scaffolds Can Enhance the Bistability of Multisite Phosphorylation Systems
Source: PLoS Comput Biol. 2012 Jun 21;8(6):e1002551. doi: 10.1371/journal.pcbi.1002551 (PMC3380838; doi:10.1371/journal.pcbi.1002551)
Supplement: Text S1 — In this supplementary text we provide more information on the mathematical analysis of the various models involved. In Section 1, we describe the concept of deficiency including a precise statement of the Deficiency Zero Theorem, then proceed to prove several of the theorems stated in the Results section of the manuscript. We also describe and apply another tool used to prove the non-existence of bistability, the concept of sign determined systems. In Section 2 we characterize the steady states of each of the four models as the solutions of two and three dimensional algebraic equations. In Section 3 we provide a detailed mathematical analysis of the idea that as , the MA systems (with or without scaffold) are approximated by the respective LR models. (PDF) [file pcbi.1002551.s004.pdf]

Protein scaffolds can enhance the multistability of multisite phosphorylation systems Carlo Chan, Xinfeng Liu, Liming Wang, Lee Bardwell, Qing Nie, and Germán Enciso

Text S1: Supplementary Information

Contents

1 Network Deficiency and Monostable Reaction Networks **1**
- 1.1 Network deficiency . . . 1
- 1.2 Applications of network deficiency . . . 2
- 1.3 Strongly sign determined systems . . . 3
- 1.4 Applications of strongly sign determined systems . . . 3
- 1.5 Equal scaffold binding rates across phosphoforms . . . 5

2 Steady State Analysis **6**
- 2.1 Model LR-NS . . . 6
- 2.2 Model NMA-S . . . 6
- 2.3 Model LR-S . . . 8
- 2.4 Model MA-S . . . 8

3 MA Approaches LR as $k_M \to \infty$ **9**
- 3.1 Constructing conditions such that LR-NS and NMA-S have equivalent steady states 10
- 3.2 Constructing conditions such that LR-S and MA-S have equivalent steady states . . 12

1 Network Deficiency and Monostable Reaction Networks

In this section, we provide details of the proofs to rule out multistability for certain scaffold binding models with linear (de)phosphorylation rates, i.e. for models of the type LR-S. We also state in detail various definitions and the main theorem of network deficiency theory and an important result regarding strongly sign determined systems.

1.1 Network deficiency

The network deficiency theory [3] can be used to prove that certain chemical reaction networks can not exhibit multistability regardless of the reaction parameters used. This creates a strong analytic result without the use of Monte Carlo simulations. We state the definitions and main theorems for network deficiency below.

A **chemical reaction network**, $\{\mathcal{S}, \mathcal{G}, \mathcal{R}\}$, consists of three sets: a finite set $\mathcal{S}$ representing the molecular species, the set of complexes $\mathcal{G}$, and the set of reactions in the network $\mathcal{R}$. We reserve the symbol $\mathcal{C}$ to denote the number of complexes in a given network. The complexes in the system are simply the left or right hand sides of every chemical reaction, e.g. $A + B$ and C for the reaction $A + B \to C$. The basic mathematical object to be studied here is a graph whose edges are the different complexes (each appearing only once), and where the directed edges are the different reactions. Notice that no mention is made of the parameters for the system, e.g. the rates at which the reactions take place. This is because the results will be independent of their (positive) values.

The **linkage classes** of a network are the strongly connected components of the undirected form of the reaction graph. That is, two complexes are in the same linkage class if there is an undirected path connecting them. We call ℓ the number of linkage classes. The **rank** of a reaction network

(denoted by r) is defined as the rank of the standard stoichiometry matrix of the system. The deficiency of a network $\{\mathcal{S}, \mathcal{G}, \mathcal{R}\}$ is defined by

$$\delta = \mathcal{C} - \ell - r$$

Say that a complex y **ultimately reacts to** z if there is a *directed* path on the reaction graph from y to z, and denote it by $y \Rightarrow z$. A chemical reaction network is **weakly reversible** if $y \Rightarrow z$ implies $z \Rightarrow y$. That is, weak reversibility means that whenever one can reach complex z from complex y through a directed path of reactions, then one can also reach y from z through another directed path.

Theorem 1.1 (Deficiency Zero Theorem). *Let $\{\mathcal{S}, \mathcal{G}, \mathcal{R}\}$ be any reaction network of deficiency zero. If the network is weakly reversible, then for any mass action kinetics $k \in (\mathbb{Z}^+)^{|\mathcal{R}|}$, the differential equations for the mass action system $\{\mathcal{S}, \mathcal{G}, \mathcal{R}, k\}$ have the following properties: there exists within each positive stoichiometric compatibility class precisely one equilibrium; that equilibrium is asymptotically stable, and there cannot exist a nontrivial cyclic composition trajectory in $(\mathbb{Z}^+)^{|\mathcal{R}|}$.*

The original proofs of this theorem date back to the work of Horn and Jackson [7] and Feinberg and Horn [4], according to Gunawardena [6]. To understand the concept of a stoichiometric compatibility class, think of a system with several forms of the same protein, say $B_0, \ldots, B_n$ for different forms of a given protein B. Suppose the total amount of substrate is constant throughout, e.g. $B_1 + \ldots + B_n = B_{tot}$. A stoichiometric compatibility class is the set of all states that have a specific total amount of B, say $B_{tot} = 100$, as well as specific total amounts of every other protein involved.

1.2 Applications of network deficiency

We apply the Deficiency Zero Theorem to show that the chemical reaction networks below cannot exhibit multistability regardless of the reaction parameters used. As stated above, we will work with the linear rate model LR-S throughout.

Theorem 1.2. *If the phosphorylation and dephosphorylation only take place for the scaffold unbound substrates, we can rule out multistability.*

Reaction Diagram:

$$B_0 \rightleftharpoons B_1 \rightleftharpoons B_2 \rightleftharpoons \ldots \rightleftharpoons B_n$$

$$B_i + S \rightleftharpoons B_iS \text{ for } i = 0, ..n$$

Proof. The reaction network is weakly reversible and we verify that

$$\begin{aligned} \mathcal{C} &= 3(n+1) \\ l &= n+2 \\ r &= 2n+1 \\ \delta &= 0 \end{aligned}$$

Hence by the Zero Deficiency theorem, there exists exactly one asymptotically stable steady state which can calculated from the steady state analysis. □

Theorem 1.3. *If the phosphorylation and dephosphorylation only take place for the scaffold bound substrates, we can rule out multistability.*

Proof. The network is weakly reversible and unlike that in the previous result, it has a single linkage class as shown in the following network:

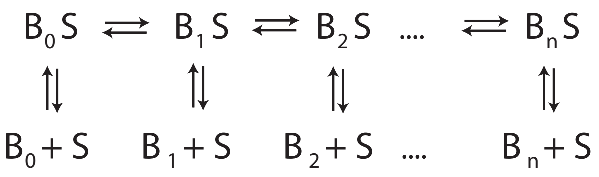

We compute the deficiency of this systems as follows:

$$\begin{aligned} \mathcal{C} &= 2(n+1) \\ l &= 1 \\ r &= 2n+1 \\ \delta &= 0 \end{aligned}$$

Hence by the Zero Deficiency theorem, there exists exactly one asymptotically stable steady state as before, for a given stoichiometric compatibility class. □

1.3 Strongly sign determined systems

For chemical reaction networks that are not weakly reversible or don't have deficiency zero, the Deficiency Zero Theorem cannot be applied. We can rule out multistability for some reaction networks by examining the stoichiometric matrix of the chemical reaction network and applying the results from [1, 2]. We state the following definitions and theorems below. Moreover ERNEST [8], a Matlab toolbox, can be used to computationally verify the results.

A chemical reaction system with n species and m reactions can be written in the form

$$x' = Mv(x),$$

where $x = [x_1, ..., x_n]^T$ is the nonnegative n-vector of species concentrations, $v = [v_1, ..., v_m]^T$ is the m-vector of the reaction rates, and M is the $n \times m$ stoichiometric matrix. A reaction system is **nonautocatalytic** if the stoichiometric matrix M and the matrix V^T, defined by the $m \times n$ matrix $V(x)$, $V_{ij} = \frac{\partial v_i}{\partial x_j}$, have opposite sign structures in the following sense: $M_{ij}V_{ji} \leq 0$ for all i, j, and $M_{ij} = 0 \to V_{ji} = 0$. In general, the assumption that a system is nonautocatalytic holds for mass action systems, provided that the reactant only occurs on one side of a reaction.

A square matrix P is **sign-nonsingular** if the sign of its determinant is nonzero and can be determined from the signs of its entries (i.e. if Q is any other matrix with $sign(p_{ij}) = sign(q_{ij})$ for all i, j, then $sign(\det P) = sign(\det Q)$). A matrix M is **strongly sign determined** if all square submatrices of M are either sign-nonsingular or singular.

Theorem 1.4. *If the reactions in a continuous flow stirred tank reactor are nonautocatalytic, and the stoichiometric matrix M is strongly sign determined, then the system does not admit multiple equilibria.*

For the original proof of this theorem see Corollary 3.5 in Banaji et al [1].

1.4 Applications of strongly sign determined systems

Theorem 1.5. *Suppose there is only one phosphorylation site, i.e. $n = 1$. If phosphorylation and dephosphorylation take place on both scaffold bound and unbound substrates then we can rule out multistability.*

Proof. This network, as well as all other networks considered in this manuscript, lie within the framework of reactions in a cotinuous flow stirred tank reactor. The network for this system is reproduced below:

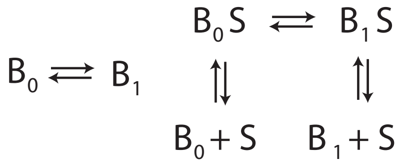

It can be verified that this system is weakly reversible, but it has deficiency 1, hence the Deficiency Zero Theorem doesn't apply. However we compute the stoichiometric matrix for $x = [B_0, B_1, B_0S, B_1S, S]^T$ as

$$M = \begin{pmatrix} -1 & 1 & 0 & 0 & 0 \\ 1 & -1 & 0 & 0 & 0 \\ 0 & 0 & 1 & -1 & 0 \\ 0 & 0 & -1 & 1 & 0 \\ -1 & 0 & 1 & 0 & -1 \\ 1 & 0 & -1 & 0 & 1 \\ 0 & -1 & 0 & 1 & -1 \\ 0 & 1 & 0 & -1 & 1 \end{pmatrix}$$

Since each species appears at most on one of the two sides of a given reaction, the system is nonautocatalytic. It was verified computationally that the stoichiometric matrix is also strongly sign determined, by inspecting each square submatrix. Using the results stated above we conclude the proof.

□

Theorem 1.6. *Suppose that there is only one phosphorylation site, phosphorylation takes place only for scaffold bound substrates and dephosphorylation takes place only on scaffold unbound substrates. Then multistability is not possible, for any reaction parameter values on a given stoichiometric compatibility class.*

Proof. This system is not weakly reversible, hence deficiency results do not apply. Once again, for $x = [B_0, B_1, B_0S, B_1S, S]^T$ the stoichiometric matrix is

$$M = \begin{pmatrix} 1 & -1 & 0 & 0 & 0 \\ 0 & 0 & -1 & 1 & 0 \\ -1 & 0 & 1 & 0 & -1 \\ 1 & 0 & -1 & 0 & 1 \\ 0 & -1 & 0 & 1 & -1 \\ 0 & 1 & 0 & -1 & 1 \end{pmatrix}$$

After checking that the system is nonautocatalytic, and after verifying numerically that M is strongly sign determined, the result follows.

□

Theorem 1.7. *For the system LR-S, suppose that there is only one phosphorylation site, phosphorylation takes place for scaffold unbound substrates and dephosphorylation takes place only on scaffold bound substrates. Then multistability is not possible, for any reaction parameter values on a given stoichiometric compatibility class.*

$$B_0 \longrightarrow B_1$$

$$B_0S \longleftarrow B_1S$$

$$B_i + S \rightleftharpoons B_iS \text{ for } i = 0, 1$$

Proof. This system is also not weakly reversible. Once again, for $x = [B_0, B_1, B_0S, B_1S, S]^T$ the stoichiometric matrix is

$$M = \begin{pmatrix} -1 & 1 & 0 & 0 & 0 \\ 0 & 0 & 1 & -1 & 0 \\ -1 & 0 & 1 & 0 & -1 \\ 1 & 0 & -1 & 0 & 1 \\ 0 & -1 & 0 & 1 & -1 \\ 0 & 1 & 0 & -1 & 1 \end{pmatrix}$$

After checking that the system is nonautocatalytic, and after verifying numerically that M is strongly sign determined, the result follows. □

1.5 Equal scaffold binding rates across phosphoforms

Theorem 1.8. *In Model LR-S, if the rates of binding and unbinding to the scaffold are independent of the phosphorylation state of the system, then multistability is not possible.*

Proof. For $i = 1, .., n-1$, we have the following rate equations:

$$\begin{aligned}
\frac{d[B_0]}{dt} &= -\alpha_0[B_0] + \delta_0[B_1] + k^d[B_0S] - k^a[B_0][S] \\
\frac{d[B_i]}{dt} &= \delta_i[B_{i+1}] + \alpha_{i-1}[B_{i-1}] - (\alpha_i + \delta_i)[B_i] + k^d[B_iS] - k^a[B_i][S] \\
\frac{d[B_n]}{dt} &= \alpha_{n-1}[B_{n-1}] - \delta_{n-1}[B_n] + k^d[B_nS] - k^a[B_n][S] \\
\frac{d[B_0S]}{dt} &= -\alpha_0[B_0S] + \delta_0[B_1S] - k^d[B_0S] + k^a[B_0][S] \\
\frac{d[B_iS]}{dt} &= \delta_i[B_{i+1}S] + \alpha_{i-1}[B_{i-1}S] - (\alpha_i + \delta_i)[B_iS] - k^d[B_iS] + k^a[B_i][S] \\
\frac{d[B_nS]}{dt} &= \alpha_{n-1}[B_{n-1}S] - \delta_{n-1}[B_nS] - k^d[B_nS] + k^a[B_n][S]
\end{aligned}$$

Let

$$\begin{aligned}
[B] &= \sum_{i=0}^{n}[B_i] \\
[BS] &= \sum_{i=0}^{n}[B_iS]
\end{aligned}$$

Then

$$\frac{d[B]}{dt} = \sum_{i=0}^{n}\frac{d[B_i]}{dt} = k^d\sum_{i=0}^{n}[B_iS] - k^a[S]\sum_{i=0}^{n}[B_i] = k^d[BS] - k^a[B][S]$$

$$\frac{d[BS]}{dt} = \sum_{i=0}^{n}\frac{d[B_iS]}{dt} = -k^d\sum_{i=0}^{n}[B_iS] + k^a[S]\sum_{i=0}^{n}[B_i] = -k^d[BS] + k^a[B][S]$$

Notice that this is the description of the much simpler system $S + B \leftrightarrow SB$, with on-rate k^a, and off-rate k^d. At steady state, we define $k = \frac{k^a}{k^d}$ then

$$[BS] = \frac{k^a}{k^d}[B][S] = k[B][S]$$

From the conservation of substrates,

$$\begin{aligned}
[B] &= B_{tot} - [BS] \\
[BS] &= S_{tot} - [S]
\end{aligned}$$

Substituting in the previous equation and simplifying, we have

$$k[S]^2 + k(B_{tot} - S_{tot} + 1)[S] - S_{tot} = 0$$

a polynomial of degree 2. Since the discriminant is positive, $(k(B_{tot} - S_{tot}) + 1)^2 + 4kS_{tot} > 0$, and the constant term is negative, there must exist exactly one positive root. Thus, there exists exactly one steady state of the reduced system, which attracts all solutions.

We still need to show that the original system has a unique, globally attractive solution. We know from our previous analysis that at steady state the free scaffold variable S is equal to a uniquely defined value S_0. Therefore after setting all left hand sides of the system to zero, we can also set $S = S_0$. But these are now the equations of a linear system, more precisely, the linear reaction system $B_i \to B_{i+1}$, $B_{i+1} \to B_i$, $SB_{i+1} \to SB_i$, $SB_i \to B_i$, and $B_i \to SB_i$, the last with rate $k^a S_0$. This linear system has a unique steady state for the given total protein concentrations, therefore the set of equations has a unique solution and the proof of the statement is complete.

□

2 Steady State Analysis

Given a multisite phosphorylation system it is sometimes possible to write all system variables at steady state explicitly in terms of chemical parameters and total protein concentration, in particular ensuring the uniqueness of a steady state solution. When this is not possible, we have followed and generalized a technique described in [9], in order to write a small system of algebraic equations involving E_{tot}, B_{tot}, and, in here, S_{tot}. The solutions of this small system of equations are in bijective correspondence with the roots of the ODE of the chemical reaction system, thus substantially reducing the problem of determining how many roots the system has. We carry out this technique with the various models involved in this paper.

2.1 Model LR-NS

Using linear rates, the model in which no scaffold is present and phosphorylation and dephosphorylation occurs on the substrates.

Let $[X^{(1)}]$ be the steady state of substrate X for model LR-NS.
Conservation equations:

$$B_{tot} = \sum_{i=0}^{n} [B_i^{(1)}]$$

Steady state concentrations: For $\zeta_i = \frac{\alpha_i}{\delta_i}$, and $A = \frac{E_{tot}}{F_{tot}}$, we have

$$[B_{i+1}^{(1)}] = \frac{\alpha_i}{\delta_i} \frac{E_{tot}}{F_{tot}} [B_i^{(1)}] = \zeta_i A [B_i^{(1)}] \text{ for } i = 0, .., n-1$$

Steady state solution: Using the conservation equation and steady state concentrations, we can solve for the steady state solution in terms of $[B_0^{(1)}]$ by

$$[B_0^{(1)}] = \frac{B_{tot}}{1 + \sum_{i=1}^{n-1} (\prod_{k=0}^{i-1} \zeta_k) A^i}$$

which can be solved explicitly for any A.

2.2 Model NMA-S

Using mass action kinetics, the model in which no scaffold is present and phosphorylation and dephosphorylation occurs on the substrates.

Let $[X^{(2)}]$ be the steady state of substrate X for model NMA-S.
Conservation equations:

$$\begin{aligned}
B_{tot} &= \sum_{i=0}^{n}[B_i^{(2)}] + \sum_{i=0}^{n-1}[EB_i^{(2)}] + \sum_{i=1}^{n}[FB_i^{(2)}] \\
E_{tot} &= [E^{(2)}] + \sum_{i=0}^{n-1}[EB_i^{(2)}] \\
F_{tot} &= [F^{(2)}] + \sum_{i=1}^{n}[FB_i^{(2)}]
\end{aligned}$$

For $i = 0, 1, ..., n-1$, the Michaelis-Menten constants are defined as follows

$$k_{M,i}^E = \frac{b_i^E + c_i}{a_i^E}$$
$$k_{M,i+1}^F = \frac{b_{i+1}^F + d_{i+1}}{a_{i+1}^F}$$

Steady state concentrations: For $i = 0, 1, ..., n-1$, $\lambda_i = \frac{c_i k_{M,i+1}^F}{d_{i+1} k_{M,i}^E}$, and $t = \frac{[E^{(2)}]}{[F^{(2)}]}$

$$\begin{aligned}
[B_{i+1}^{(2)}] &= \frac{c_i k_{M,i+1}^F}{d_{i+1} k_{M,i}^E} \frac{[E^{(2)}]}{[F^{(2)}]}[B_i^{(2)}] = \lambda_i t [B_i^{(2)}] \\
[EB_i^{(2)}] &= \frac{1}{k_{M,i}^E}[E^{(2)}][B_i^{(2)}] \\
[FB_{i+1}^{(2)}] &= \frac{1}{k_{M,i+1}^F}[F^{(2)}][B_{i+1}^{(2)}]
\end{aligned}$$

Then for $i = 1, ..., n$, we have

$$[B_i^{(2)}] = \prod_{k=0}^{i-1} \lambda_k t^i [B_0^{(2)}]$$

Steady state solution: We introduce the polynomials, $\phi_0^{(2)}(t), \phi_1^{(2)}(t), \phi_2^{(2)}(t)$ defined below

$$\begin{aligned}
\sum_{i=0}^{n}[B_i^{(2)}] &= [B_0^{(2)}](1 + \sum_{i=1}^{n}(\prod_{k=0}^{i-1}\lambda_k)t^i = [B_0^{(2)}]\phi_0^{(2)}(t) \\
\sum_{i=0}^{n-1}[EB_i^{(2)}] &= [E^{(2)}][B_0^{(2)}](\sum_{i=0}^{n-1}(\frac{1}{k_{M,i}^E}\prod_{k=0}^{i-1}\lambda_k)t^i = [E^{(2)}][B_0^{(2)}]\phi_1^{(2)}(t) \\
\sum_{i=1}^{n}[FB_i^{(2)}] &= [B_0^{(2)}]\sum_{i=1}^{n}(\frac{1}{k_{M,i}^F}\prod_{k=0}^{i-1}\lambda_k)t^i = [F^{(2)}][B_0^{(2)}]\phi_2^{(2)}(t)
\end{aligned}$$

Using the conservation equations, we have

$$\begin{aligned}
B_{tot} &= [B_0^{(2)}](\phi_0^{(2)}(t) + [E^{(2)}]\phi_1^{(2)}(t) + [F^{(2)}]\phi_2^{(2)}(t)) \\
E_{tot} &= [E^{(2)}](1 + [B_0^{(2)}]\phi_1^{(2)}(t)) \\
F_{tot} &= [F^{(2)}](1 + [B_0^{(2)}]\phi_2^{(2)}(t))
\end{aligned}$$

Solving for $[B_0^{(2)}]$, we define a the steady state equation as a function $\Phi^{(2)} : \mathbb{R}_+^2 \to \mathbb{R}^2$ by

$$\begin{aligned}\Phi_1^{(2)}([E^{(2)}],[F^{(2)}]) &= [E^{(2)}]\left(1+\frac{\phi_1^{(2)}(t)B_{tot}}{\phi_0^{(2)}(t)+[E^{(2)}]\phi_1^{(2)}(t)+[F^{(2)}]\phi_2^{(2)}(t)}\right)-E_{tot}\\ \Phi_2^{(2)}([E^{(2)}],[F^{(2)}]) &= [F^{(2)}]\left(1+\frac{\phi_2^{(2)}(t)B_{tot}}{\phi_0^{(2)}(t)+[E^{(2)}]\phi_1^{(2)}(t)+[F^{(2)}]\phi_2^{(2)}(t)}\right)-F_{tot}\end{aligned}$$

where the roots of $\Phi^{(2)}$ are the steady state solutions for NMA-S.

2.3 Model LR-S

Using linear rates, the model in which phosphorylation takes place on and off scaffold and dephosphorylation only occurs off scaffold.

Let $[X^{(1s)}]$ be the steady state of substrate X for model LR-S.
Conservation equations:

$$B_{tot} = \sum_{i=0}^{n}([B_i^{(1s)}]+[B_iS^{(1s)}])$$

$$S_{tot} = [S^{(1s)}]+\sum_{i=0}^{n}[B_iS^{(1s)}]$$

Steady state concentrations:

$$[B_i^{(1s)}]+[B_iS^{(1s)}] = \frac{\alpha_{i-1}E_{tot}}{\delta_iF_{tot}}[B_iS^{(1s)}] = \lambda_{i-1}A[B_iS^{(1s)}] \text{ for } i=1,...,n$$

We need to set $[B_0S^{(1s)}] = f([S^{(1s)}])\cdot[B_0^{(1s)}]$, where $f:\mathbb{R}_+\to\mathbb{R}$. This is done by recursive solving the following equations from the highest index to the lowest.
For $i=1,...,n-1$, let

$$\begin{aligned}\eta_i &= \frac{\delta_i}{k_i^d}\\ \beta_{i+1} &= \frac{\delta_{i+1}}{k_i^d}\\ \kappa_i &= \frac{k_n^a}{k_n^d}\end{aligned}$$

We have

$$\begin{aligned}[B_nS^{(1s)}] &= (\eta_iF_{tot}+\delta_n[S^{(1s)}])[B_n^{(1s)}]\\ \beta_{i+1}F_{tot}[B_{i+1}^{(1s)}]+[B_iS^{(1s)}] &= (\eta_iF_{tot}]+\delta_i[S^{(1s)}])[B_i^{(1s)}] \text{ for } i=n-1:-1:1\\ \beta_1F_{tot}[B_1^{(1s)}]+[B_0S^{(1s)}] &= \beta_0[B_0^{(1s)}][S^{(1s)}]\end{aligned}$$

using this recursively, we can find the function f such that $[B_0S^{(1s)}] = f([S^{(1s)}])\cdot[B_0^{(1s)}]$. Solving for the steady state equation, as in LR-NS, we see that the steady state solution reduces to solving the roots of a polynomial of degree $n+2$.

2.4 Model MA-S

Using mass action kinetics, the model in which phosphorylation takes place on and off scaffold and dephosphorylation only occurs off scaffold.

Conservation equations:

$$
\begin{aligned}
B_{tot} &= \sum_{i=0}^{n}([B_i^{(2s)}] + [B_iS^{(2s)}]) + \sum_{i=0}^{n-1}[EB_iS^{(2s)}] + \sum_{i=1}^{n}([FB_i^{(2s)}] + [FB_iS^{(2s)}]) \\
S_{tot} &= \sum_{i=0}^{n}[B_iS^{(2s)}] + \sum_{i=0}^{n-1}[EB_iS^{(2s)}] \\
E_{tot} &= [E^{(2s)}] + \sum_{i=0}^{n-1}[EB_iS^{(2s)}] \\
F_{tot} &= [F^{(2s)}] + \sum_{i=1}^{n}([FB_i^{(2s)}] + [FB_iS^{(2s)}])
\end{aligned}
$$

Steady state concentrations:

$$
\begin{aligned}
[EB_iS^{(2s)}] &= \frac{1}{k_{M,i}^E}[E^{(2s)}][B_iS^{(2s)}] \text{ for } i = 0, ..., n-1 \\
[FB_i^{(2s)}] &= \frac{1}{k_{M,i}^F}[F^{(2s)}][B_i^{(2s)}] \text{ for } i = 1, ..., n \\
[FB_iS^{(2s)}] &= \frac{1}{k_{M,i}^F}[F^{(2s)}][B_iS^{(2s)}] \text{ for } i = 1, ..., n \\
[B_i]^{(2s)} + [B_iS^{(2s)}] &= \frac{c_{i-1}k_{M,i}^F}{d_ik_{M,i}^E}\frac{[E^{(2s)}]}{[F^{(2s)}]}[B_iS^{(2s)}] = \lambda_{i-1}t[B_iS^{(2s)}] \text{ for } i = 1, ..., n
\end{aligned}
$$

We need to set $[B_0S^{(2s)}] = f([E^{(2s)}], [F^{(2s)}], [S^{(2s)}]) \cdot [B_0^{(2s)}] \cdot [S^{(2s)}]$, where $f : \mathbb{R}_+^3 \to \mathbb{R}$. This is done by recursive using the following equations, below, from the highest to the lowest index.
For $i = 1, ..., n-1$, let

$$
\begin{aligned}
\eta_i &= \frac{d_i}{k_i^d k_{M,i}^F} \\
\beta_{i+1} &= \frac{d_{i+1}}{k_i^d k_{M,i+1}^F} \\
\kappa_i &= \frac{k_n^a}{k_n^d}
\end{aligned}
$$

Then

$$
\begin{aligned}
[B_nS^{(2s)}] &= (\eta_n[F^{(2s)}] + \kappa_n[S^{(2s)}])[B_n^{(2s)}] \\
\beta_{i+1}[F^{(2s)}][B_{i+1}^{(2s)}] + [B_iS^{(2s)}] &= (\eta_i[F^{(2s)}] + \kappa_i[S^{(2s)}])[B_i^{(2s)}] \text{ for } i = n-1 : -1 : 1 \\
\beta_1[F^{(2s)}][B_1^{(2s)}] + [B_0S^{(2s)}] &= \beta_0[B_0^{(2s)}][S^{(2s)}]
\end{aligned}
$$

Using this recursively, we can find the function f such that $[B_0S^{(2s)}] = f([E^{(2s)}], [F^{(2s)}], [S^{(2s)}]) \cdot [B_0^{(2s)}] \cdot [S^{(2s)}]$.

3 MA Approaches LR as $k_M \to \infty$

For simplicity in the proof, we revert to the model in which phosphorylation only takes place on scaffold and dephosphorylation only occurs off scaffold. The addition of dephosphorylation on scaffold components will result in model LR-S and MA-S which is a similar model in terms of the construction of the steady state equation, and determining multistability. We refer to both models as LR-S and MA-S, respectively. It is stated in the main text that as k_M becomes large, intuitively the system NMA-S starts to resemble LR-NS (and similarly, MA-S resembles LR-S). In this section we make that statement more precise, by describing the convergence of one model towards the other in detail.

3.1 Constructing conditions such that LR-NS and NMA-S have equivalent steady states

For $i = 0, .., n-1$, let β_i^E,$\beta_{i+1}^F < O(\frac{1}{\epsilon})$ such that

$$\frac{1}{k_{M,i}^E} = \beta_i^E \epsilon$$
$$\frac{1}{k_{M,i+1}^F} = \beta_{i+1}^F \epsilon$$

Since $\lambda_i = \bar{\lambda_i}$,

$$\frac{\alpha_i}{\delta_{i+1}} = \frac{c_i k_{M,i+1}^F}{d_{i+1} k_{M,i}^E} = \frac{c_i \beta_i^E}{d_{i+1} \beta_{i+1}^F}$$

Then given α_i, δ_{i+1}, for arbitrary d_{i+1} or c_i

$$\begin{aligned} c_i &= d_{i+1} \frac{\alpha_i \beta_{i+1}^F}{\delta_{i+1} \beta_i^E} \text{ or} \\ d_{i+1} &= c_i \frac{\delta_{i+1} \beta_i^E}{\alpha_i \beta_{i+1}^F} \end{aligned}$$

Then for

$$\frac{1}{k_{M,i}^E} = \frac{a_i^E}{b_i^E + c_i} = \beta_i^E \epsilon$$
$$\frac{1}{k_{M,i+1}^F} = \frac{a_{i+1}^F}{b_{i+1}^F + d_{i+1}} = \beta_{i+1}^F \epsilon$$

For arbitrary b_i^E or a_i^E, we have

$$\begin{aligned} a_i^E &= \beta_i^E \epsilon (b_i^E + c_i) \text{ or} \\ b_i^E &= \frac{a_i^E}{\beta_i^E \epsilon} - c_i (> 0 \text{ provided } \epsilon \text{ is small enough}) \end{aligned}$$

Similarly for b_{i+1}^F or a_{i+1}^F,

$$\begin{aligned} a_{i+1}^F &= \beta_{i+1}^F \epsilon (b_{i+1}^F + d_{i+1}) \text{ or} \\ b_{i+1}^F &= \frac{a_{i+1}^F}{\beta_{i+1}^F \epsilon} - d_{i+1} (> 0 \text{ provided } \epsilon \text{ is small enough}) \end{aligned}$$

We define k_M as

$$k_M = \min_{i=0,\ldots,n-1} \{k_{M,i}^E, k_{M,i+1}^F\}$$

Lemma 3.1. *If $k_M \gg 1$ then $[E^{(2)}] \approx E_{tot}$ and $[F^{(2)}] \approx F_{tot}$.*

Proof. Let $\frac{1}{k_M} = O(\epsilon)$.
For $i = 0, ..., n-1$, using the conservation and steady states equations, we have

$$\begin{aligned} \frac{E_{tot} - [E^{(2)}]}{E_{tot}} &= \frac{\sum_{i=0}^{n-1} [EB_i^{(2)}]}{E_{tot}} = \frac{[E^{(2)}]}{E_{tot}} \sum_{i=0}^{n-1} \frac{1}{k_{M,i}^E} [B_i^{(2)}] \le n B_{tot} \frac{1}{k_M} = O(\epsilon) \\ \frac{F_{tot} - [F^{(2)}]}{F_{tot}} &= \frac{\sum_{i=1}^{n} [FB_i^{(2)}]}{F_{tot}} = \frac{[F^{(2)}]}{F_{tot}} \sum_{i=1}^{n} \frac{1}{k_{M,i}^F} [B_i^{(2)}] \le n B_{tot} \frac{1}{k_M} = O(\epsilon) \end{aligned}$$

Then $[E^{(2)}] \to E_{tot}$ and $[F^{(2)}] \to F_{tot}$ as $\epsilon \to 0$. □

Theorem 3.2. *If $\frac{1}{k_M} \ll 1$, then we can construct parameters such that $[B_i^{(2)}] \approx [B_i^{(1)}]$ for all $i = 0, 1, ..., n$.*

Proof. Let $\frac{1}{k_M} = O(\epsilon)$.
Using the conservation equation of substrate at steady state, we have

$$\begin{aligned} B_{tot} &= \sum_{i=0}^{n}[B_i^{(2)}] + \sum_{i=0}^{n-1}\frac{1}{k_{M,i}^E}[E^{(2)}][B_i^{(2)}] + \sum_{i=1}^{n}\frac{1}{k_{M,i}^F}[F^{(2)}][B_i^{(2)}] \\ &= \sum_{i=0}^{n}[B_i^{(2)}] + \frac{1}{k_M}\Big(\sum_{i=0}^{n-1}[E^{(2)}][B_i^{(2)}] + \sum_{i=1}^{n}[F^{(2)}][B_i^{(2)}]\Big) \\ &= [B_0^{(2)}]\Big(1 + \sum_{i=1}^{n-1}\Big(\prod_{k=0}^{i-1}\lambda_k\Big)t^i + O(\epsilon)\Big) \end{aligned}$$

If B_{tot} is the same for both models and

$$\lambda_i = \zeta_i \text{ for } i = 0, 1, ..., n$$

and solving for the steady states of NMA-S,

$$[B_0^{(2)}] = \frac{B_{tot}}{1 + \sum_{i=1}^{n}\Big(\prod_{k=0}^{i-1}\lambda_k\Big)t^i + O(\epsilon)}$$

then

$$|[B_0^{(2)}] - [B_0^{(1)}]| = \frac{B_{tot}}{M}\left|\sum_{i=1}^{n}\prod_{k=0}^{i-1}\lambda_k(u^i - A^i)\right| = \frac{B_{tot}}{M}\max_{i=1,...,n}\left\{\prod_{k=0}^{i-1}\lambda_k\right\}\sum_{i=0}^{n}(t^i - A^i) \to 0$$

where $M = (1+\sum_{i=1}^{n-1}(\prod_{k=0}^{i-1}\lambda_k)u^i + O(\epsilon))(1+\sum_{i=1}^{n-1}(\prod_{k=0}^{i-1}\lambda_k A^i)$ and by Lemma 6.1. Thus for any $i = 1, ..., n$

$$|[B_i^{(2)}] - [B_i^{(1)}]| = \prod_{k=0}^{i-1}\lambda_k|u^i[B_0^{(2)}] - A^i[B_0^{(1)}]| \to 0$$

since $u \to A$ and $[B_0^{(2)}] \to [B_0^{(1)}]$ as $\epsilon \to 0$. □

Theorem 3.3. *If $E_{tot}, F_{tot} \gg B_{tot}$ then $[E^{(2)}] \approx E_{tot}$ and $[F^{(2)}] \approx F_{tot}$.*

Proof.

$$\begin{aligned} \frac{E_{tot} - [E^{(2)}]}{E_{tot}} &= \frac{\sum_{i=0}^{n-1}[EB_i^{(2)}]}{E_{tot}} \le \frac{B_{tot}}{E_{tot}} = O(\epsilon) \\ \frac{F_{tot} - [F^{(2)}]}{F_{tot}} &= \frac{\sum_{i=0}^{n-1}[FB_i^{(2)}]}{F_{tot}} \le \frac{B_{tot}}{F_{tot}} = O(\epsilon) \end{aligned}$$

□

Remark 3.4. *Note that if $E_t, F_t \gg B_{tot}$, it does not necessarily imply that $[B_{(i)}^{(2)}] \approx [B_{(i)}^{(1)}]$ for all i, though both models will exhibit monostability [5].*

Suppose $[B_i] = [B_i^{(1)}] = [B_i^{(2)}]$ for all $i = 0,..,n$.
Let $\frac{1}{k^E_{M,i}}, \frac{1}{k^F_{M,i+1}} = 1$ for all $i = 0, 1, ..., n-1$. Then for LR-NS, the conservation equation is

$$B_{tot} = \sum_{i=0}^{n} [B_i]$$

At steady state, the conservation equation for model NMA-S is

$$\begin{aligned} B_{tot} &= \sum_{i=0}^{n} [B_i] + \sum_{i=0}^{n-1} [EB_i^{(2)}] + \sum_{i=1}^{n} [FB_i^{(2)}] \\ &= \sum_{i=0}^{n} [B_i] + \sum_{i=0}^{n-1} \frac{1}{k^E_{M,i}} [E^{(2)}][B_i] + \sum_{i=1}^{n} \frac{1}{k^F_{M,i}} [F][B_i] \\ &= (1 + [E^{(2)}])[B_0] + (1 + [E^{(2)}] + [F^{(2)}]) \sum_{i=0}^{n-1} [B_i] + (1 + [F^{(2)}])[B_n] \end{aligned}$$

Subtracting the two equations, we have

$$0 = [E^{(2)}][B_0] + ([E^{(2)}] + [F^{(2)}]) \sum_{i=0}^{n-1} [B_i] + [F^{(2)}][B_n]$$

Since $B_{tot} \neq 0$, there exists at least one $i \in 0, 1, ..., n$ such that $[B_i] \neq 0$. Suppose $[B_0] \neq 0$ then $[E^{(2)}] = 0$ but $[E^{(2)}] \approx E_{tot}$ from Theorem 6.3. Similarly for $[B_n] \neq 0$ then $[F^{(2)}] = 0$ and $[B_i] \neq 0$ for $i \in \{1, ..., n-1\}$ then $[E^{(2)}] = [F^{(2)}] = 0$.

3.2 Constructing conditions such that LR-S and MA-S have equivalent steady states

For $i = 0,..,n-1$, let β_i^E,$\beta_{i+1}^F < O(\frac{1}{\epsilon})$ then

$$\begin{aligned} c_i &= \frac{\alpha_i}{\beta_i^E \epsilon} \\ d_{i+1} &= \frac{\delta_{i+1}}{\beta_{i+1}^F \epsilon} \end{aligned}$$

Then for arbitrary b_i^E or a_i^E

$$\begin{aligned} a_i^E &= \beta_i^E \epsilon (b_i^E + c_i) = \beta_i^E \epsilon (b_i^E + \frac{\alpha_i}{\beta_i^E \epsilon}) = \epsilon \beta_i^E b_i^E + \alpha_i \text{ or} \\ b_i^E &= \frac{a_i^E - \alpha_i}{\beta_i^E \epsilon} \text{ provided } a_i^E > \alpha_i. \end{aligned}$$

Similarly for arbitrary b_{i+1}^F or a_{i+1}^F,

$$\begin{aligned} a_{i+1}^F &= \beta_{i+1}^F \epsilon (b_{i+1}^F + d_{i+1}) = \beta_{i+1}^F \epsilon (b_{i+1}^F + \frac{\delta_{i+1}}{\beta_{i+1}^F \epsilon}) = \epsilon \beta_{i+1}^F b_{i+1}^F + \delta_{i+1} \text{ or} \\ b_{i+1}^F &= \frac{a_{i+1}^F - \delta_{i+1}}{\beta_{i+1}^F \epsilon} \text{ provided } a_{i+1}^F > \delta_{i+1}. \end{aligned}$$

Lemma 3.5. *If $\frac{1}{k_M} \ll 1$ then $[E^{(2s)}] \approx E_{tot}$ and $[F^{(2s)}] \approx F_{tot}$.*

Proof. Let $\frac{1}{k_M} = O(\epsilon)$.
For $i = 0, ..., n-1$, using the conservation and steady states equations, we have

$$\begin{aligned}
\frac{E_{tot} - [E^{(2s)}]}{E_{tot}} &= \frac{\sum_{i=0}^{n-1}[EB_iS^{(2s)}]}{E_{tot}} = \frac{[E^{(2s)}]}{E_{tot}} \sum_{i=0}^{n-1} \frac{1}{k^E_{M,i}} [B_i^{(2s)}] \leq nB_{tot}\frac{1}{k_M} = O(\epsilon) \\
\frac{F_{tot} - [F^{(2s)}]}{F_{tot}} &= \frac{\sum_{i=1}^{n}[FB_i^{(2s)}]}{F_{tot}} = \frac{[F^{(2s)}]}{F_{tot}} \sum_{i=1}^{n} \frac{1}{k^F_{M,i}} [B_i^{(2s)}] \leq nB_{tot}\frac{1}{k_M} = O(\epsilon)
\end{aligned}$$

Then $[E^{(2s)}] \to E_{tot}$ and $[F^{(2s)}] \to F_{tot}$ as $\epsilon \to 0$. □

Theorem 3.6. *If* $\frac{1}{k_M} \ll 1$, *then we can construct parameters such that* $[B_i^{(2s)}] \approx [B_i^{(1s)}]$ *and* $[B_iS^{(2s)}] \approx [B_iS^{(1s)}]$.

Proof. By Lemma 6.5, $[E^{(2s)}] \to E_{tot}$ and $[F^{(2s)}] \to F_{tot}$.
For $i = 0, 1, ..., n-1$, let

$$\begin{aligned}
c_i &= \alpha_i k^E_{M,i} \\
d_{i+1} &= \delta_{i+1} k^F_{M,i+1}
\end{aligned}$$

Using the steady state equations and letting $A = \frac{E_{tot}}{F_{tot}}$ we have

$$\begin{aligned}
[B_i^{(2s)}] &= = \lambda_i \frac{[E^{(2s)}]}{[F^{(2s)}]}[B_{i-1}S^{(2s)}] \to \lambda_i A [B_{i-1}S^{(2s)}] \text{ for } i = 1, .., n \\
[B_0S^{(2s)}] &= \frac{k_0^a}{\alpha_0[E^{(2s)}] + k_0^d}[B_0^{(2s)}][S^{[2s]}] \to \frac{k_0^a}{\alpha_0 E_{tot} + k_0^d}[B_0^{(2s)}][S^{(2s)}] \\
[B_iS^{(2s)}] &= \frac{k_i^a}{\alpha_i[E^{(2s)}] + k_i^d}[B_i^{(2s)}][S^{(2s)}] + \frac{\alpha_{i-1}[E^{(2s)}]}{\alpha_i[E^{(2s)}] + k_i^d}[B_{i-1}S^{(2s)}] \\
&\to \frac{k_i^a}{\alpha_i E_{tot} + k_i^d}[B_i^{(2s)}][S^{(2s)}] + \frac{\alpha_{i-1}E_{tot}}{\alpha_i E_{tot} + k_i^d}[B_{i-1}S^{(2s)}] \text{ for } i = 1, .., n-1 \\
[B_nS^{(2s)}] &= \frac{k_n^a}{k_n^d}[B_n^{(2s)}][S^{(2s)}] + \frac{\alpha_n[E^{(2s)}]}{k_n^d}[B_{n-1}S^{(2s)}] \to \frac{k_n^a}{k_n^d}[B_n^{(2s)}][S^{(2s)}] + \frac{\alpha_n E_{tot}}{k_n^d}[B_{n-1}S^{(2s)}] \\
[EB_iS^{(2s)}] &\to 0 \text{ for } i = 0, ..., n-1 \\
[FB_i^{(2s)}] &\to 0 \text{ for } i = 1, ..., n
\end{aligned}$$

Thus, the construction of the steady state equation to solve for $[S^{(2s)}]$ will reduce to the same polynomial as in LR-S. □

References

[1] M. Banaji, P. Donnell, and S. Baigent. *p* matrix properties, injectivity, and stability in chemical reaction systems. *SIAM J. Appl. Math.*, 67(6):1523–1547, 2007.

[2] G. Craciun and M. Feinberg. Multiple equilibria in complex chemical reaction networks: Semiopen mass action systems. *SIAM J. Appl. Math.*, 70(6):18591877, 2010.

[3] M. Feinberg. Chemical reaction network structure and the stability of complex isothermal reactors– I. the deficiency zero and deficiency one theorems. *Chem. Eng. Sci.*, 42(10):2229 – 2268, 1987.

[4] M. Feinberg and F. Horn. Chemical mechanism structure and the coincidence of the stoichiometric and kinetic subspace. *Proc. Nat. Acad. Sci. USA*, 66:83-97, 1977.

[5] J. Gunawardena. Multisite protein phosphorylation makes a good threshold but can be a poor switch. *Proc. Nat. Acad. Sci. USA*, 102(41):1461714622, 2005.

[6] J. Gunawardena. Chemical reaction network theory for *in silico* biologists. Lecture Notes, Bauer Center for Genomics Research, Harvard University, Cambridge MA, 2003.

[7] F. Horn and R. Jackson. General mass action kinetics. *Arch. Rat. Mech. Anal.*, 47:81-116, 1972.

[8] N. Soranzo and C. Altafini. ERNEST: a toolbox for chemical reaction network theory. *Bioinformatics*, 25(21):28532854, 2009.

[9] M. Thomson and J. Gunawardena. Unlimited multistability in multisite phosphorylation systems. *Nature*, 460(7252):274–7, 2009.
